# Intensifying Nutrient Removal in Hybrid-Constructed Wetlands Treating Urban Streamwater

**DOI:** 10.1021/acsomega.4c10124

**Published:** 2025-04-02

**Authors:** André Gustavo Patel, Débora Gonçalves Bortolini, Adelania de Oliveira Souza, Mateus Xavier de Lima, Ana Paula Trevisan, Vsevólod Mymrin, André Nagalli, Fernando Hermes Passig, Karina Querne de Carvalho

**Affiliations:** † Federal University of Technology − Paraná (UTFPR) - Civil Engineering Graduate Program, Deputado Heitor de Alencar Furtado St., 5000, Ecoville, Curitiba, Paraná 81.280-340, Brazil; ‡ Federal University of Technology − Paraná (UTFPR) − Environmental Sciences and Technology Graduate Program, Deputado Heitor de Alencar Furtado St., 5000, Ecoville, Curitiba, Paraná 81.280-340, Brazil; § Western Paraná State University (UNIOESTE) - Agricultural Engineering Graduate Program, Universitária St., 2069, Jardim Universitário, Cascavel, Paraná 85.819-110, Brazil; ∥ Federal University of Technology − Paraná (UTFPR). Civil Construction Academic Department, Deputado Heitor de Alencar Furtado St., 5000, Ecoville, Curitiba, Paraná 81.280-340 Brazil; ⊥ Federal University of Technology − Paraná (UTFPR) − Chemistry and Biology Academic Department, Deputado Heitor de Alencar Furtado St., 5000, Ecoville, Curitiba, Paraná 81280-340, Brazil

## Abstract

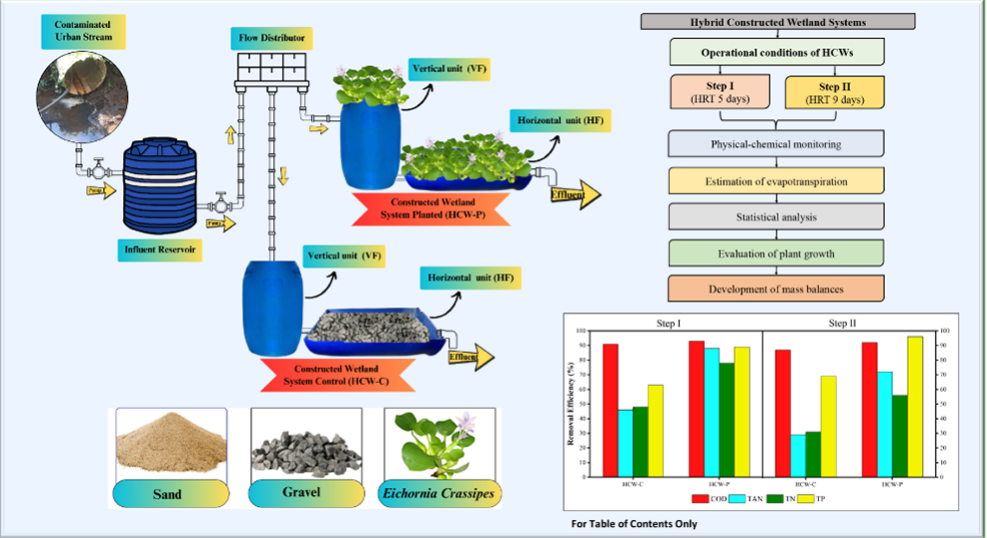

This study investigated the influence of hydraulic retention time (HRT) variation and the presence of macrophytes on the efficiency of two pilot-scale hybrid-constructed wetlands (HCWs) treating urban streamwater contaminated with nontreated sanitary sewage contributions from the surrounding communities. Each HCW comprises a vertical unit (VF) and a horizontal unit (HF) filled with sand and gravel. HCW-P was planted with*Eichornia crassipes* onto the filtering media, and HCW-C was set up as a control unit with no macrophytes. The novelty of this study consists of evaluating the combination of these factors (HRT and macrophytes) in the operation of HCWs for removing organic matter and nutrients. The operation of the HCWs was divided into step I, with a hydraulic retention time (HRT) of 9 days for 133 days, and step II, with an HRT of 5 days for 131 days. Neither HRT variation (p-value = 0.7691) nor the presence of macrophytes (*p*-value = 0.0941) influenced the COD removal, as the HCWs achieved high removal efficiencies (>87%) during the operation. HCW-P achieved higher total nitrogen (TN) removal efficiencies in steps I and II (56% and 78%) compared to HCW-C (31% and 48%) during the operation, demonstrating the improvement in removing TN due to the presence of macrophytes (p-value ≤ 0.05). In addition, the shorter HRT promoted an increase of 22% in TN removal for HCW-P (p-value ≤ 0.05). The macrophytes and longer HRT enhanced total ammonia nitrogen (TAN) removal, as HCW-P (46% and 88%) achieved higher removal efficiencies than HCW-C (29% and 72%) in steps I and II, respectively (*p*-value ≤ 0.05). Regarding total phosphorus (TP), HCW-C and HCW-P achieved removal efficiencies of 63% and 89% in step I and 69% and 96% in step II, confirming the influence of HRT and macrophytes on TP removal. Finally, macrophytes demonstrated adaptability and resilience to the operational conditions, even when fixed in HCWs, which presented robustness in removing organic matter and nutrients from the urban streamwater via biofilm assimilation and adsorption under HRT variations.

## 1 Introduction

Natural urban streams have been continuously polluted by various human activities, such as exacerbated urban growth, irregular land use, and inadequate urban planning, which increases the inappropriate discharge of wastewater into water bodies^1,2^ due to insufficient implementation and/or maintenance of basic sanitation infrastructure. Wastewater, commonly rich in organic matter, nutrients, and emerging contaminants, can be classified depending on its source and characteristics as gray wastewater,^3^ sanitary sewage,^4^ industrial effluents,^5^ and leachate,^6^ among others. Regardless of the classification, it can lead to eutrophication, harm the life of aquatic animals and spread diseases to humans and animals by pathogens.^7,8^

Constructed wetlands (CW) stand out as an example of nature-based technology combined with engineering practices to achieve high efficiency in removing organic matter and nutrients in wastewater treatment, creating and restoring habitats, and improving the esthetics of the urban environment. These cost-effective and sustainable systems are simple to design and operate, require little or no energy, and are resistant to variations in organic and hydraulic loading rates, treating different types of wastewater, including urban streamwater.^9,10^

The combination of different constructed wetland configurations, such as vertical flow (VFCW), horizontal subsurface flow (HFCW), and surface flow (SFCW) units, composes hybrid-constructed wetlands (HCWs). These innovative systems can reduce the limitations of these units when applied individually,^11,12^ achieving average removal efficiencies of 57–75% for COD, 30–80% for TN, 22–62% for TAN, and 34–61% for TP^13^ in the treatment of synthetic wastewater,^14^ sanitary sewage,^15^ industrial effluents,^16^ and livestock wastewater.^17^

However, studies applying HCWs in the treatment of urban streamwater are still necessary to better understand their applicability and feasibility (costs of implementation and maintenance), limitations (clogging and available area), potential (operation), optimization (physicochemical parameters, hydraulic loading rate—HLR, and hydraulic retention time—HRT), and mechanisms involved.^18^ Various studies have reported different mechanisms that occur in CWs, such as biodegradation for organic matter removal; adsorption, ammonification, nitrification, denitrification, ammonia volatilization, anaerobic ammonia oxidation, and plant and microbial assimilation for nitrogen removal; plant and substrate (media) adsorption and precipitation for phosphorus removal; and phytoremediation, complexation, precipitation, and microbial oxidation/reduction for metal removal.^19−21^

Several species of macrophytes have been applied in CWs depending on the system configuration. In our study, we selected the floating macrophyte *Eichornia crassipes*, also known as water hyacinth, an abundant and native species that presents fast growth^22^ and easy adaptation to climatic conditions (temperature, humidity, and precipitation), capable of removing micropollutants^23^ and nutrients,^24^ and with phytoremediation potential.^25^ These characteristics reinforce the capability of this free-floating species to minimize contamination and toxicity,^26^ even when planted in constructed wetlands.^27^

Additionally, the filtering materials in CWs can influence plant growth, as observed by Mello et al.,^24^ who demonstrated higher TN and TP removal using *E. crassipes* and the most effective COD removal in a planted HCW using sand and gravel, and by Lima et al.,^23^ who explored the adaptability of *E. crassipes* in different substrates under subtropical conditions in vertical-flow constructed wetlands. The authors revealed increased vegetal density in the units filled with bricks, light-expanded clay aggregates (LECA), and gravel, demonstrating the robustness, adaptability, and reproducibility of this species in diverse filtering materials.

Another important factor is the effect of HRT on the removal of contaminants in CWs, since longer HRT could improve the removal efficiency, mainly for nitrogen and organic matter. Extended contact time between water and filtering media tends to enable more opportunities for adsorption and microbial transformation processes, such as nitrification and denitrification, which are vital for nutrient removal. Conversely, longer HRT may also increase operational costs due to greater land requirements and risks of nutrient accumulation in the system.^12,28^

Considering these facts, this study evaluated the influence of*Eichornia crassipes* and the variation of the hydraulic retention time (HRT, from 9 to 5 days) on the performance of a hybrid constructed wetland (VF-HF CW) filled with sand and gravel in the treatment of a natural urban stream. Moreover, nitrogen phytoextraction by*E. crassipes*, evapotranspiration influence, and identification of the main mechanisms were also investigated.

## 2 Material and Methods

### 2.1 Location

This investigation was performed in Curitiba, Paraná, Brazil, where the climate is temperate and subtropical, without a defined dry period. The annual average temperature varies from 10 to 22 °C, with annual precipitation of 1,450 mm and relative humidity of 80.5%.^29^ The experiment was conducted at the Federal University of Technology—Paraná in Curitiba, at 935 m above sea level (5°26′39” S, 49°21′16” W), to treat the water of a contaminated urban stream that crosses the campus, receiving sewage contributions from the surrounding community with characteristics of low-strength sewage according to the classification of Metcalf and Eddy,^30^ who defined COD values below 339 mg L^–1^ as “weak” sewage.

### 2.2 Construction of Wetlands Systems

**Figure 1:**
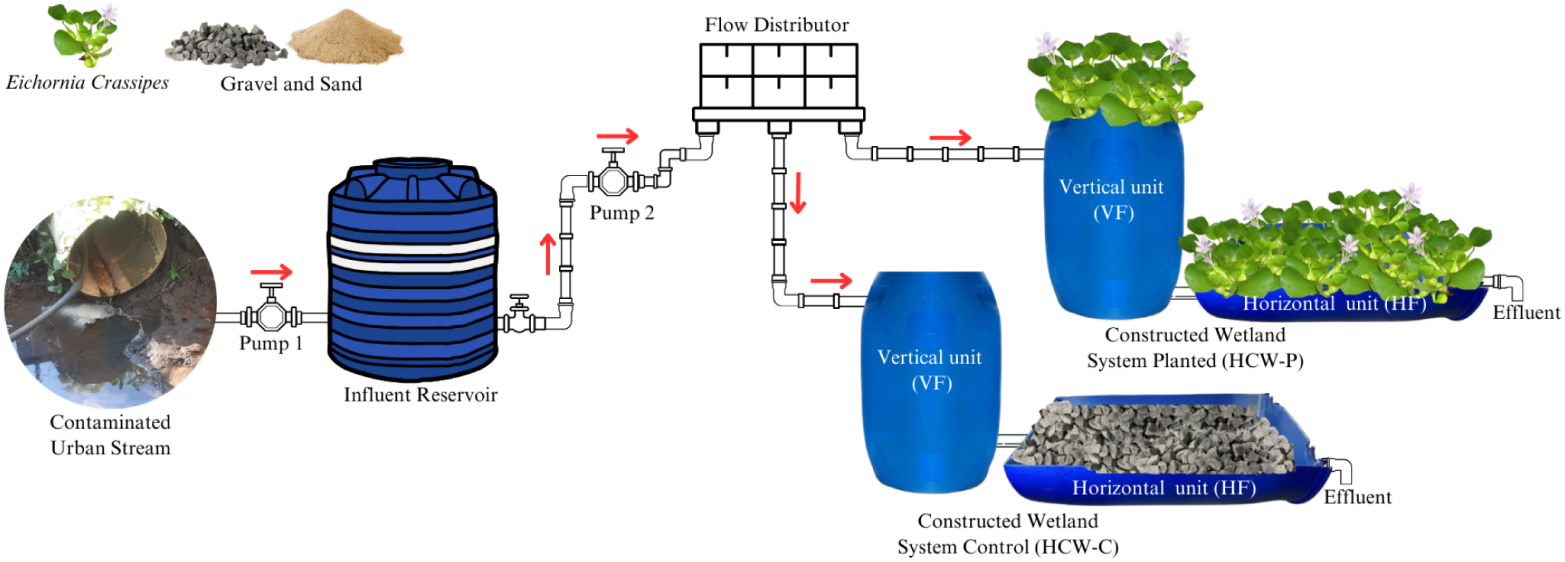
Scheme of the constructed wetlands systems. Note: Photo taken by MSc. André Gustavo Patel.

Two pilot-scale hybrid constructed wetland systems (HCW) were built, consisting of a vertical unit followed by a horizontal unit, both filled with gravel no 1 (granulometry varying between 9.5 and 19 mm) and sand layers (Figure 1). HCW-P was planted with macrophyte *Eichhornia crassipes*nto the filtering media of the vertical and horizontal units. HCW-C was built as a control system with no macrophytes planted on the vertical and horizontal units. The useful volume of the units totaled 0.146 m^3^ in each HCW system (Figure S1).

Table 1 describes the dimensions of the subsuperficial systems built with cylindrical polyethylene containers.

**Table 1:** Dimensions of the Constructed Wetlands Systems

Characteristics	Vertical unit	Horizontal unit
Height (m)	0.88	0.46
Diameter (m)	0.56	0.56
Radius (m)	0.28	0.28
Surface area (m^2^)	0.246	0.378
Number of seedlings/plants (un)	18	23
Vegetation density (plants m^–2^)	90	115
Height of gravel layer (m)	0.30	0.20
Granulometry of gravel no. 1 (mm)	9.5–19	9.5–19
Height of sand layer (m)	0.40	0.21
Sand granulometry (mm)	2–4	2–4
Height of gravel layer at the bottom (m)	0.10	0.05
Total volume (m^3^)	0.197	0.191
Useful volume (m^3^)	0.074	0.072

Seedlings of the macrophyte*Eichhornia crassipes*, popularly known as Water Hyacinth, were collected at the Parque Náutico in Curitiba. In the laboratory, the macrophytes were continuously washed with tap water to remove coarse materials and transferred to polypropylene reservoirs containing urban streamwater. After 2 weeks of adaptation, the macrophytes were transplanted onto the filtering materials of the HCW-P. *E. crassipes* was chosen because it is a native species with fast growth, phytoremediation potential, and adaptability to climate variations.^31−33^ This species is being successfully cultivated in the filtering media of CWs, even though it is a free-floating species. During the operation of HCW-P, no replacement or cutting of macrophytes was carried out.

Samples of the influent (urban streamwater) and effluent (treated) of the hybrid systems were collected by a set of polyvinyl chloride (PVC) pipes of 25 mm in diameter, 20 cm in length, and 20 cm in width, perforated with orifices of 5 mm. Additionally, samples of the influent and effluent of the vertical and horizontal units (planted and control) were collected. The continuous feeding was carried out through a submersible pump (FERRARI, XKS-401 PW), which transported the water from the stream to a reservoir and then to a flow distributor for each hybrid system. The working volume was 0.146 m^3^, which sums both units.

The operation of HCW-C and HCW-P was divided into two steps: I, with a flow rate of 0.7 L h^–1^ and a hydraulic retention time (HRT) of 9 days for 133 days; and II, with a flow rate of 1.2 L h^–1^ and an HRT of 5 days for 131 days. The loading rates (LR) were calculated by the product of each pollutant concentration in the influent and effluent samples (mg L^–1^) by the influent flow rate (L d^–1^), divided by the superficial area of each system (m^2^).

### 2.3 HCW Monitoring Through Physical and Chemical Analysis

The subsuperficial systems were monitored weekly by determining temperature (°C) (thermometer), pH (4500-H^+^ B), oxidation–reduction potential—ORP (mV) (2580 ORP), turbidity (2130_B), chemical oxygen demand—COD (mg L^–1^) (5220 D) in raw samples, total Kjeldahl nitrogen—TKN (mg L^–1^) (4500-Norg macro Kjeldahl), total ammonia nitrogen—TAN (mg L^–1^) (4500-NH_3_–N C), nitrite (mg L^–1^) (4500-NO_2_^–^ B), nitrate (mg L^–1^) (4500-NO_3_^–^ dimethylphenol), and total phosphorus—TP (mg L^–1^) (4500-P I) in samples of the influent and effluent of the systems, following the procedures of the American Public Health Association (APHA).^34^ Total alkalinity was determined according to Ripley et al.^35^ All the parameters were determined in duplicate (*n* = 32) during 151 days.

The removal efficiency (RE; %) was determined by considering the difference between the loading rates (LR; mg m^–2^ d^–1^) of the influent and effluent samples, as shown in the following calculation formula:
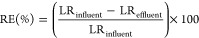
1

The chemicals used in the physical and chemical analyses were purchased from Merck (Germany). Temperature and pH were determined by using a Quimis Q400AS pHmeter (Brazil). ORP and turbidity were measured with a YSI PRO 10 (USA) meter and a Policontrol AP2000 W turbidimeter (Brazil). TKN and TAN were determined using a digestor/distillator analyzer, Velp Scientifica UDK 159 (Italy). COD, N–NO_2_^–^, N–NO_3_^–^, and TP were determined using a spectrophotometer, Hach Company DR5000 (USA).

### 2.4 Plant Uptake of Total Nitrogen and Total Phosphorus

At the end of each experimental phase, plant samples were dried in an oven with air recirculation at 65 °C for 72 h and triturated in a Wiley mill. Posteriorly, total nitrogen (TN) and total phosphorus (TP) were quantified in the dry matter of stems and leaves following Empresa Brasileira de Pesquisa Agropecuária (EMBRAPA, 1999)^36^ and Pompêo and Moschini-Carlos,^37^ respectively.

Total nitrogen (TNSS) and total phosphorus (TPSS) standing stocks were calculated considering the nutrient concentration multiplied by mass production and divided by the surface area.

### 2.5 Evapotranspiration

Evapotranspiration was estimated by monitoring the weight of bench-scale polyethylene containers of 11 cm width × 20 cm length × 11.5 cm height, totaling 2.53 L in volume for each container. The containers were filled with filtering materials in proportion to those adopted in the HCW systems. Twelve containers were used, half planted (group HCW-P) and half unplanted (group HCW-C). In each group, three containers represented the vertical units, and the other three represented the horizontal units, simulating the hybrid systems.

The containers were fed weekly in batch mode with 500 mL of water from the contaminated urban stream (the same used for feeding the HCW systems). The weight of each container was measured immediately after feeding and again after 24 h. The evapotranspiration rate was calculated according to Celis,^38^ as shown in the following formula.
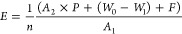
2

whereas *E* = average evaporation (mm d^–1^); *n* = number of days in 1 period; *A*_1_ = internal area of the container (mm^2^); *A*_2_ = average area of the container (mm^2^); *p* = daily precipitation (mm d^–1^); *W*_0_ = mass of the container at time 0 (initial mass; g); *W*_1_ = mass of the container at time 1 (final mass; g); *F* = weight added to or removed from the container (g).

### 2.6 Statistical Analysis

Kolmogorov–Smirnov and Shapiro–Wilk tests (*p*-value <0.05) were applied to compare the performance of the HCW systems. The nonparametric Mann–Whitney U test (p-value <0.05) was used to evaluate data for which normal distribution was not verified.

Statistical analyses were performed using the free software BioStat, version 5.3, with 95% confidence.

## 3 Results and Discussion

### 3.1 Hybrid Constructed Wetlands Monitoring

Table 2 presents the physical and chemical characterization of the water from the contaminated urban stream (influent) and the effluent of the HCW systems. During the study, the temperature in the influent varied from 18 to 24 °C in step I and from 17 to 21 °C in step II.

**Table 2:** Monitoring Parameters of Hybrid Constructed Wetlands Systems^a^

Step I														
	Influent				HCW-C					**HCW-P**				
Parameters	X	SD	Min	Max	X	SD	Min	Max	E%	X	SD	Min	Max	E%
***T***	22	2	18	24	22	2	18	24	-	22	2	18	24	-
**pH**	7.8	0.3	7.4	8.2	7.8	0.2	7.5	8.0	-	7.6	0.2	7.3	7.8	-
**ORP**	403	91	215	529	448	63	294	489	-	464	33	410	498	-
**TA**	160	40	119	228	144	40	111	237	-	169	17	150	199	-
**Turbidity**	12.7	2.7	8.6	18.2	1.5	0.3	1.0	2.2	-	0.4	0.1	0.3	0.5	-
**COD**	85	16	34	133	8	1	2	21	91	4	0.4	2	7	93
**TN**	76	16	56	97	47	13	31	61	48	20	4	9	49	78
**TAN**	62	8	49	73	33	9	6	55	46	7	1	2	18	88
**N-NO**_**2**_^**–**^	0.221	0.010	0.036	0.852	0.067	0.010	0.015	0.109	49	0.062	0.010	0.039	0.100	48
**N-NO**_**3**_^**–**^	0.042	0.010	0.034	0.048	0.040	0.010	0.032	0.052	10	0.040	0.010	0.033	0.056	14
**TP**	3.09	0.45	0.32	5.53	1.16	0.010	0.08	1.96	63	0.22	0.030	0.08	0.29	89

Step II														
	Influent				HCW-C					HCW-P				
Parameters	X	SD	Min	Max	X	SD	Min	Max		X	SD	Min	Max	
***T***	19	1	17	21	18	1	16	21	-	18	1	16	20	-
**pH**	7.8	0.3	7.3	8.2	7.8	0.2	7.4	8.1	-	7.5	0.7	6.3	8.0	-
**ORP**	422	68	333	552	423	31	385	476	-	423	40	369	487	-
**TA**	149	43	77	193	134	36	78	174	-	144	37	71	198	-
**Turbidity**	19.0	4.2	13.0	26.9	1.2	0.4	0.6	1.8	-	0.03	0.01	0.02	0.03	-
**COD**	111	14	71	138	15	1	3	36	87	8	1	1	16	92
**TN**	65	16	45	97	44	7	36	55	31	28	8	14	46	56
**TAN**	51	11	41	70	36	7	21	45	29	15	3	3	33	72
**N-NO**_**2**_^**–**^	0.110	0.003	0.062	0.169	0.039	0.002	0.003	0.096	71	0.020	0.001	0.001	0.061	79
**N-NO**_**3**_^**–**^	2.54	0.69	1.17	4.32	1.45	0.38	0.59	2.52	44	0.93	0.14	0.25	1.80	58
**TP**	12	2.0	10	15	4.0	0.63	2.0	5.0	69	0.5	0.01	0.04	2.0	96

a *X:* average (arithmetic); SD: standard deviation; Max: maximum value; Min: minimum value; *E*: removal efficiency (%); HCW-C: hybrid constructed wetland—control; HCW-P: hybrid constructed wetland—planted; *T*: liquid temperature (°C); pH: hydrogen ionic potential; ORP: oxidation–reduction potential (mV); TA: total alkalinity (mgCaCO_3_ L^–1^); turbidity (NTU); COD: chemical oxygen demand (raw samples; mg L^–1^); TN: total nitrogen (mg L^–1^); TAN: total ammonia nitrogen (mg L^–1^); N-NO_2_^–^: nitrite (mg L^–1^); N-NO_3_^–^: nitrate (mg L^–1^); TP: total phosphorus (mg L^–1^)

For both steps, the temperature ranged from 16 to 24 °C in the effluent samples, resulting in an average temperature below the optimum range suggested by Akpor et al.,^39^ which should be between 30 and 40 °C for nutrient removal. However, the temperature in the systems was above the limiting temperature of 15 °C for removing contaminants, as indicated by Papaevangelou et al.^40,41^ In addition, the temperature was close to 20 °C on some days, which may have contributed to ammonia volatilization and nitrite oxidation.^41,42^

The values obtained in our study for temperature may inhibit or limit biological treatment and denitrification since they are below the range of 25–35 °C indicated by Metcalf and Eddy,^30^ Kadlec and Wallace,^28^ and Sezerino et al.^43^ No significant differences were observed between the planted and nonplanted systems in each step (*p*-value = 0.8745 in step I; *p*-value = 0.3163 in step II); however, significant differences were noted between the steps in each system (*p*-value ≤ 0.05 for both planted and nonplanted HCW systems).

The pH values varied from 7.3 to 8.2 in influent samples in steps I and II. In step I, pH ranged from 7.5 to 8.0 in HCW-C and 7.3–7.9 in HCW-P in effluent samples. In step II, pH varied from 7.4 to 8.1 in HCW-C and from 6.3 to 8.0 in HCW-P in effluent samples (Table 2). In addition, no significant differences were observed between the samples of the planted and nonplanted systems in each step (*p*-value = 0.1031 in step I; *p*-value = 0.3717 in step II), nor between the steps in each system (*p*-value = 0.2933 planted; *p*-value = 0.5990 nonplanted), indicating that the presence of *E. crassipes* and the HRT applied did not influence the pH of the systems.

The pH results directly influence the ammonification, nitrification, and denitrification processes. According to the IWA Specialist Group on the Use of Macrophytes in Water Pollution, a pH between 7.5 and 8.6 is favorable for nitrification;^44^ however, a pH lower than 7.5 limits nitrification, and a pH above 8.5 favors ammonia volatilization.^32^ In this study, the observed pH was favorable for nitrification, and most nitrogen content was identified as ammonia (NH_3_).

The mean total alkalinity of the influent was 160 gCaCO_3_ L^–1^ for step I and 149 gCaCO_3_ L^–1^ for step II. In step I, effluent samples from HCW-C and HCW-P were statistically different (*p* ≤ 0.05); however, the effluent samples from the systems were not significantly different in step II (p-value = 0.6363). There were no significant differences in total alkalinity between steps I and II (*p*-value = 0.8335 for nonplanted; *p*-value = 0.0927 for planted). Alkalinity is an important parameter because it can favor ammonia formation. Approximately 7.1 gCaCO_3_ of alkalinity is required to nitrificate 1 g of ammonia.^35^ However, alkalinity can promote the development of anammox microorganisms at high levels, favoring the denitrification process.^45^ Therefore, monitoring alkalinity in CW systems is essential to guarantee efficient nitrogen removal.

Regarding oxidation–reduction potential (ORP), values consistently above +294 mV in influent and effluent samples of the HCW systems suggest an aerobic environment during the operation according to the classification of Kadlec and Wallace^28^ of ORP values above +100 mV for an aerobic environment. No significant differences were observed between the effluent samples of HCW-C and HCW-P (*p*-value = 0.5286; *p*-value = 0.9904) in both steps; however, significant differences were noted between steps I and II for each HCW system, indicating a more aerobic environment under the high HRT applied (*p*-value ≤ 0.05).

Turbidity varied from 8.6 to 26.9 NTU in the influent samples during the operation (Table 2). In the effluent samples of step I, turbidity ranged from 1.0 to 2.2 NTU in HCW-C and 0.3–0.5 NTU in HCW-P, resulting in average removal efficiencies of 88% and 97%, respectively. In step I (9 days), HCW-C and HCW-P achieved 88% and 99% removal efficiencies, respectively. By reducing HRT in step II (5 days), removal efficiencies improved to 90% in HCW-C and maintained 99% in HCW-P. These results demonstrate the effectiveness of the HCW systems in turbidity removal, even with variations in HRT, especially in HCW-P.

Significant differences were obtained between the effluent samples of HCW-C and HCW-P (*p*-value ≤ 0.05; *p*-value ≤ 0.05) in both steps and between steps I and II for HCW-P (*p*-value ≤ 0.05); however, no significant differences were obtained in the effluent samples when comparing both operational steps for HCW-C (*p*-value = 0.1344), thus reinforcing the role of the macrophyte in retaining the suspended solids in the effluent.

In summary, *E. crassipes* enhanced turbidity removal, and the increase in the hydraulic retention time (HRT) improved aeration, further underscoring the potential of hybrid systems in optimizing wastewater treatment systems. These results contribute to our understanding of the synergistic effects in aquatic environments and suggest practical applications for improving the efficiency of water and wastewater treatment processes.

### 3.2 Hybrid Constructed Wetland Systems Efficiency

The main parameters used to evaluate the efficiency of wastewater treatment consist of carbon concentration (as chemical oxygen demand, COD), nitrogen concentration (as total nitrogen, TN; total ammonia nitrogen, TAN; nitrite, N-NO_2_^–^; nitrate, N-NO_3_^–^; and total phosphorus, TP).

The efficiency of removing COD, nitrogen, and phosphorus compounds depends on a number of factors, as previously mentioned, such as the type of filter material, operational strategies, temperature, presence of macrophytes, the redox conditions of the medium, and the applied loading rates (LR), as highlighted by Wu et al.^46^

Total COD ranged from 34 to 138 mg L^–1^ in the influent samples during the operation, resulting in a “weak” concentration (COD < 339 mg L^–1^) following the classification proposed by Metcalf and Eddy^30^ for weak sanitary sewage. In step I (9 days), HCW-C and HCW-P achieved 91% and 93% removal efficiencies, which correspond to the average remaining concentrations of 8.0 and 4.0 mg L^–1^, respectively. The remaining concentrations correspond to the residual concentrations in the effluent samples. In step II, the systems reached average removal efficiencies of 87% and 92%, corresponding to the average remaining concentrations of 16.0 and 8.0 mg L^–1^, respectively. Statistical analysis (*n* = 32) indicated that neither HRT variation (*p*-value = 0.7691) nor the presence of macrophytes (*p*-value = 0.0941) influenced the COD removal during the operation.

**Figure 2:**
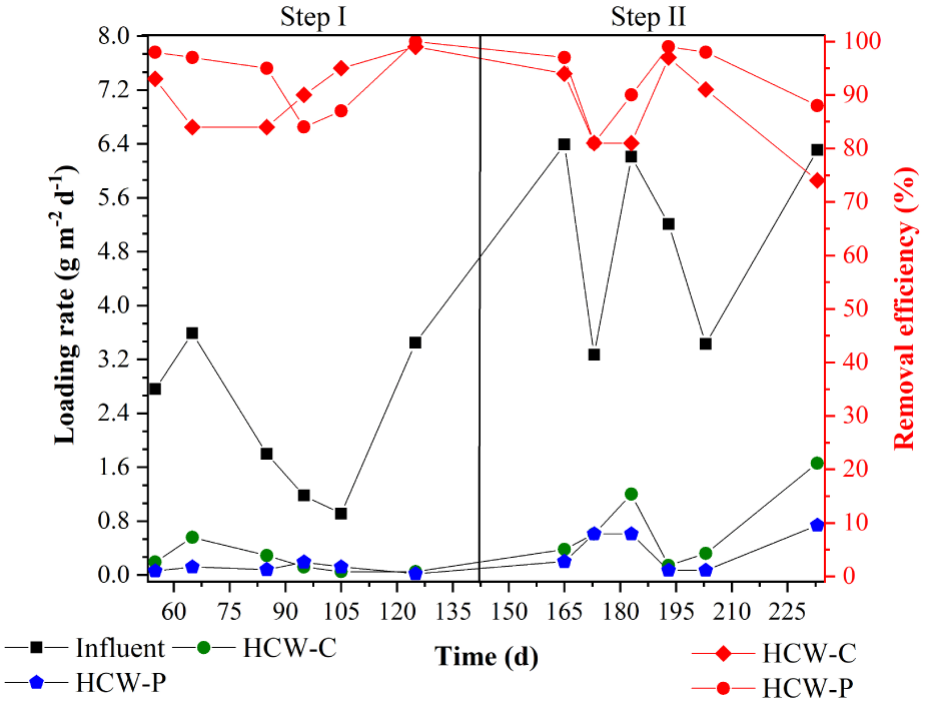
COD loading rates and removal efficiencies of the HCW systems.

Figure 2 shows that, despite the variations in the organic loading rate (OLR) applied to the systems, HCW-C and HCW-P dampened the COD variations in the urban streamwater during the study.

In step I, HCW-C and HCW-P achieved 91% and 93% removal efficiencies for the applied COD LR of 2.28 g m^–2^ d^–1^, corresponding to loading removal rates of 2.07 g m^–2^ d^–1^ and 2.19 g m^–2^ d^–1^, respectively. Regarding step II, HCW-C and HCW-P achieved loading removal rates of 4.42 g m^–2^ d^–1^ (86%) and 4.75 g m^–2^ d^–1^ (92%) for the applied COD LR of 5.14 g m^–2^ d^–1^ (Figure 2). Regarding COD removal, both systems presented steady and consistent removal performances during the entire operation period.

Singh and Vaishya^47^ evaluated a VF-HF HCW (volume of 56.82 L) planted with *Calibanus hookeri* and *Canna indica* on filtering material composed of sand, gravel, and cold drink plastic bottle chips for the treatment of municipal wastewater (COD of 546.47 mg L^–1^, LR of 144.11 g COD m^–2^ d^–1^). The authors observed the robustness of this configuration, as the HCW achieved removal efficiencies of up to 89.9% of the COD.

Hybrid-constructed wetlands on a full scale have successfully treated domestic sewage after a pretreatment stage. Gholipour and Stefanakis^48^ obtained a removal efficiency of 73.8% in HCW treating pretreated sanitary sewage (COD of 250 mg L^–1^, LR of 25 gCOD m^–2^ d^–1^) by an anaerobic baffled reactor (ABR, HRT 5.4 days). The HCW was composed of a VFCW (HRT 2.5 days) planted with *Aruno donax* followed by an HFCW (HRT 4 days) planted with *Phragmites australis* onto a compacted layer of fine sand and clay covered by a high-density polyethylene (HDPE) geomembrane. In addition, Jóźwiakowska and Bugajski^49^ verified a removal efficiency of 90% in HCW, composed of a VFCW planted with *Miscanthus giganteus*, followed by an HFCW planted with *Salix viminalis* L., both on soil, treating mechanically pretreated sanitary wastewater (COD of 336 mg L^–1^; LR of 14.93 gCOD m^–2^ d^–1^).

Our study focused on treating urban streamwater, which, despite its contamination, presents characteristics of weak sanitary sewage with no requirement for a pretreatment unit. In addition to evaluating the sole inputs and outputs within the HCW systems, our study yielded results within the ranges of these investigations. It is noteworthy that some of the referenced studies delve into the full-scale CW application, demonstrating the scalability potential implied by HCW. Thus, the results suggest a promising trajectory for the upscaling of our approach.

**Figure 3:**
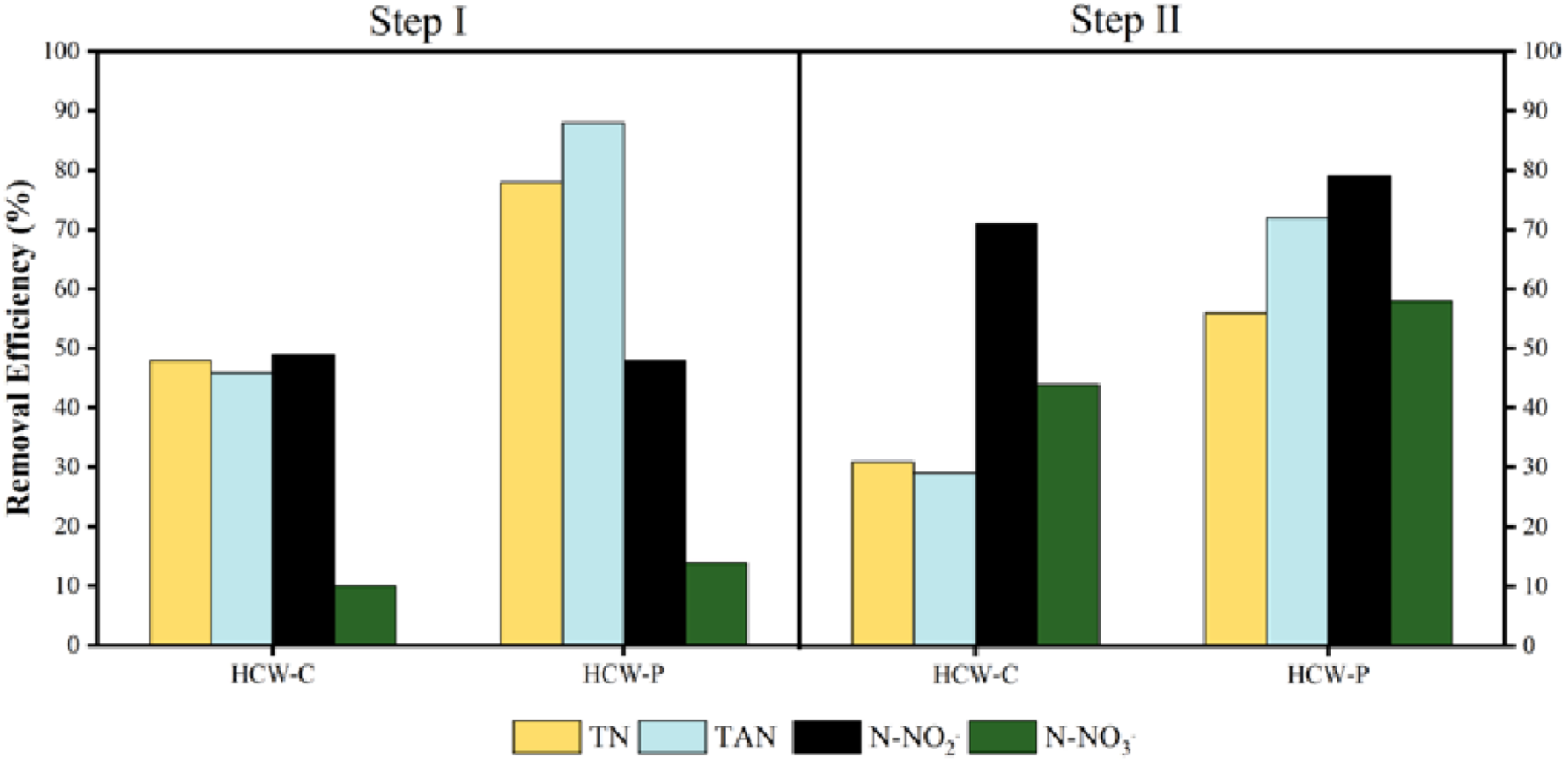
Removal efficiencies of nitrogen forms (TN, TAN, N-NO_2_^–^, and N-NO_3_^–^) of the HCW systems. Note: step I (HRT 9 d); step II (HRT 5 d); VC: vertical unit control (unplanted); HC: horizontal unit control (unplanted); VP: vertical unit planted; HP: horizontal unit planted.

Total nitrogen (TN) varied in the influent from 56 to 97 mg L^–1^ in step I (LR 2.04 g m^–2^ d^–1^) and 45–97 mg L^–1^ in step II (LR 2.98 g m^–2^ d^–1^), indicating a “medium” to “strong” concentration according to Metcalf and Eddy^30^ classification. As demonstrated in Figure 3, HCW-C and HCW-P removed 48% and 78% in step I and 31% and 56% in step II, respectively. TN concentrations remained at 47 mg L^–1^ and 20 mg L^–1^ in step I and 44 mg L^–1^ and 28 mg L^–1^ for HCW-C and HCW-P, respectively.

These results indicate that macrophytes influenced TN removal, as HCW-P achieved higher removal efficiencies in both steps (*n* = 32; p-value ≤ 0.05). No significant differences in TN removal were observed between steps I and II for HCW-C (*n* = 32; p-value = 0.7904). However, the higher HRT promoted an enhancement in TN removal for HCW-P, with high removal efficiency in step I (*n* = 32; p-value ≤ 0.05).

Similar TN removal efficiencies were observed in hybrid systems evaluated by Abbasi et al.,^50^ who verified the range of 23.8–64.8% in HCW systems planted with*Abelmoschus esculentus*,*Solanum melongena*, and*Capsicum annuum* L., respectively, using gravel and ceramsite to treat domestic wastewater with an average influent TN concentration of 28.12 (3.46) mg L^–1^. He et al.^51^ obtained 57.7% in an integrated vertical downflow-upflow CW followed by an HSF CW planted with*Canna indica* L.,*Juncus effusus* L., and*Scirpus validusVahl* onto gravel to treat domestic wastewater with an initial TN concentration of 18.9 mg L^–1^.

TAN loading rates resulted in 1.66 g m^–2^ d^–1^ and 2.37 g m^–2^ d^–1^ in the influent samples of steps I and II, respectively. Higher TAN removal efficiencies were achieved by HCW-C (46%) and HCW-P (88%) in step I with an HRT of 9 days, in comparison with the efficiencies of 29% (HCW-C) and 72% (HCW-P) observed during step II (HRT of 5 days) (Figure 3). In the effluent samples, TAN concentrations were lower than 36 mg L^–1^ in HCW-C and lower than 15 mg L^–1^ in HCW-P. These values were lower than 200 mg L^–1^ indicated by Yang et al.^52^ as toxic for plants.

Additionally, the results suggest the influence of macrophytes on TAN removal, as HCW-P achieved higher removal efficiencies than HCW-C in both steps (*n* = 32; *p*-value ≤ 0.05, Table 2). No significant differences were noted for HCW-C when operated under different HRTs in steps I and II (*n* = 32; p-value = 0.2275). The higher HRT contributed to HCW-P achieving higher removal efficiency, with a difference of 16% between steps I and II (*n* = 32; p-value ≤ 0.05), demonstrating the influence of HRT on the removal of TAN in the presence of *E. crassipes*.

Abbasi et al.^50^ observed similar TAN removal efficiencies, varying from 25% to 92.2%, in hybrid CWs planted with*A. esculentus*,*S. melongena*, and *C. annuum*L., respectively, onto gravel and ceramsite treating domestic wastewater with an influent TAN concentration of 12.62 mg L^–1^; and He et al.^51^ obtained 85.7% in an integrated vertical downflow-upflow CW followed by an HSF CW planted with*Canna indica*L.,*Juncus effusus* L., and*Scirpus validusVahl* onto gravel to treat domestic wastewater with an initial TAN concentration of approximately 10.0 mg L^–1^.

Nitrite (N-NO_2_^–^) varied in the influent from 0.036 to 0.852 mg L^–1^ in step I and 0.062–0.169 mg L^–1^ in step II, corresponding to global removal efficiencies of 49% in HCW-C and 48% in HCW-P in step I (HRT 9 d), respectively. Under lower HRT (5 d), HCW-C and HCW-P achieved higher removal efficiencies of 71% and 79% in step II, respectively, indicating that the macrophytes did not influence nitrite removal. Conversely, lower HRT increased the removal efficiencies by 22% in HCW-C and 31% in HCW-P (Figure 3).

Nitrate (N-NO_3_^–^) varied in the influent from 0.034 to 0.048 mg L^–1^ in step I and from 1.17 to 4.32 mg L^–1^ in step II. HCW-C and HCW-P achieved average global removal efficiencies of 10% and 14% in step I and 44% and 58% in step II, respectively (Figure 3). *E. crassipes* did not influence the removal of N-NO_3_^–^ in the systems operated at higher HRT (9 days); however, a difference of 14% was noted between the systems when operated at lower HRT (5 d).

**Figure 4:**
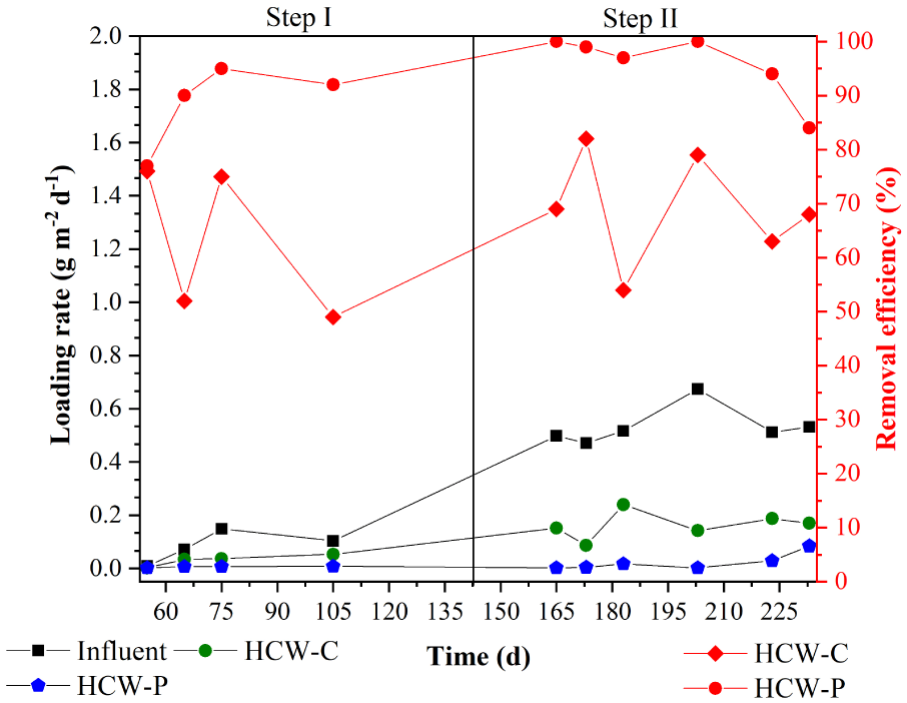
TP loading rates and removal efficiencies of the HCW systems.

Total phosphorus concentration in the influent was notably different between steps I and II (p-value ≤ 0.05), with averages of 3.09 and 12.00 mg L^–1^, corresponding to the applied loading rates of 0.08 and 0.53 g m^–2^ d^–1^, respectively, for steps I and II (Figure 4 and Table 2). These values indicated a “weak” concentration of TP in step I and a “strong” concentration in Step II, following the classification proposed by Metcalf and Eddy^30^ for sanitary sewage. The remaining loading rates resulted in 0.05 and 0.07 g m^–2^ d^–1^ in step I and 0.37 and 0.23 g m^–2^ d^–1^ in step II for HCW-C and HCW-P, respectively. The differences in total phosphorus concentrations underscore the probable influence of urban activities and, consequently, discharges of domestic wastewater daily, promoting the TP variations during the HCWs’ operation.

In step I, HCW-C and HCW-P achieved average removal efficiencies of 63% and 89%, corresponding to average remaining concentrations of 1.16 mg L^–1^ and 0.22 mg L^–1^, respectively. In step II, these systems maintained their effectiveness, achieving average removal efficiencies of 69% and 96%, corresponding to average remaining concentrations of 4.00 and 0.50 mg L^–1^, respectively. Based on the statistical analysis, the longer HRT (p-value ≤ 0.05, *n* = 32) and the presence of *E. crassipes* (p-value ≤ 0.05, *n* = 32) positively contributed to enhancing TP removal.

TP removal in constructed wetlands can occur through phosphorus assimilation as a primary nutrient for plant growth, biochemical mineralization causing sedimentation and precipitation, and filtering and adsorption effects through interaction with the substrate material.^53^

Dell’Osbel et al.^54^ observed that using plants without any filtering material is unsuitable for high levels of TP removal when operating a WWTP consisting of an anaerobic biodigester (BD), followed by a floating constructed wetland (FCW), a VFCW filled with gravel, and an HFCW filled with gravel and clay bricks. The authors verified that the TP decrease was statistically different only for the VFCW and HFCW, evidencing the importance of the filtering material and the combination of these units to achieve 95% TP removal. Therefore, the combination of mechanisms of assimilation of nutrients by plants, filtering, and nutrient adsorption onto substrates, is important for optimizing TP removal. Furthermore, these studies show that VF-HF HCW is a great and low-cost alternative for improving the removal of TP from wastewater.

Other authors observed TP removal >90% by applying VF-HF HCW. Micek et al.^4^ obtained removal efficiencies between 90% and 94% in an HCW planted with *Phragmites australis* (VF) and *Salix viminalis* L. (HF) onto chick sand, treating domestic wastewater with an initial TP concentration of 5 mg L^–1^. Moreover, Saeed et al.^55^ achieved a removal efficiency higher than 98% in an HCW planted with *Phragmites* and *Vetiver* onto stone and gravel, treating municipal wastewater with TP concentrations varying from 18.2 to 32 mg L^–1^. Thus, the results obtained in our study corroborate with TP removal in VF-HF HCW as described in the literature.

### 3.3 Evaluation of Plant Growth

In our study, the growth of *E. crassipes* was monitored by observing the number of plants developed in the vertical and horizontal units of HCW-P during the operation, as shown in Table 3. In the vertical unit, the number of plants increased by 50%, leading to a 52% increase in vegetal density. Similarly, the horizontal unit shows a 27.7% increase in the number of plants, resulting in a higher vegetal density of 45.2%. These findings suggest that *E. crassipes* readily adapted to the HCW environment. Notably, the number of plants in the horizontal unit was lower than that in the vertical unit, possibly due to the lower nutrient availability in the horizontal unit.

**Table 3:** Vegetal Density in the HCW-P

	Before treatment		After treatment (264 days)	
System	Number of plants	Density of plants (plants m^–2^)	Number of plants	Density of plants (plants m^–2^)
Vertical	12	48	18	73
Horizontal	18	73	23	106

In addition, the efficiency of nutrient plant uptake could be evaluated by analyzing TN and TP contents in vegetal tissues, commonly in leaves, petioles, and roots. Roots were probably responsible for nutrient assimilation since they presented more abundant biomass than other tissues (Table 4). The increased amount and density of macrophytes could be due to the supply of phosphate, ammonia, nitrite, and nitrate, which are generally bioavailable forms of these plant nutrients.^56^

**Table 4:** Nitrogen and Phosphorus Content in Plant Biomass

System	Parts of the plants	TN (mg g^–1^)	TP (mg g^–1^)
Vertical	Roots	70.75	26.91
	Petioles	52.31	10.76
	Leaves	55.53	6.71
	Total	59.53	44.39
Horizontal	Roots	65.01	30.95
	Petioles	43.69	18.20
	Leaves	39.89	15.23
	Total	49.53	21.46

Dell’Osbel et al.^54^ observed similar TN values in the leaves of *Canna x generalis* (>20 mg g^–1^),*Chrysopogon zizanioides* (>15 mg g^–1^), and*Xanthosoma violaceum* (35 mg g^–1^) in a VFCW and*C. generalis* (>20 mg g^–1^) and*Cyperus papyrosnanus* (>25 mg g^–1^) in an HFCW treating urban wastewater. These authors also found that TP accumulated in the leaves of these species, with approximately 10 mg g^–1^ in the VFCW and more than 8.0 mg g^–1^ in the HFCW.

TNSS concentrations resulted in 11.77 g m^–2^ in the vertical unit and 6.12 g m^–2^ in the horizontal unit. TPSS concentrations resulted in 10.37 g m^–2^ in the vertical unit and 6.92 g m^–2^ in the horizontal unit of the HCW-P. These results were higher than the range verified by Kumwimba et al.^57^ of 0.96–7.01 gTN m^–2^ and 0.21–1.41 gTP m^–2^ for*I. sibirica*, and 0.70–5.04 gTN m^–2^ and 0.08–0.43 gTP m^–2^ for*M. aquaticum*, respectively.

These results further reinforce the role of plants in enhancing the nutrient removal efficiency within the system, thus corroborating the potential use of *E. crassipes* in hybrid wetlands.

### 3.4 Evapotranspiration

Evapotranspiration is water loss resulting from the evaporation of water bodies and plant transpiration.^58^ In our study, temperature and seasonal variations influenced evapotranspiration in hybrid-constructed wetlands (Table 5). The observed differences in water loss between steps I and II (p-value ≤ 0.05) could be attributed to the variations in temperature (p-value ≤ 0.05).

**Table 5:** Evapotranspiration (Mm d^–1^) in the Hybrid Constructed Wetland Systems^a^

**Step I**										
	HCW-C					HCW-P				
	X	DP	CV	Min	Max	X	DP	CV	Min	Max
Vertical	2.12	0.01	0.11	2.11	2.12	2.58	0.01	0.09	2.57	2.58
Horizontal	2.12	0.01	0.11	2.12	2.13	2.58	0.01	0.09	2.57	2.58

**Step II**										
	HCW-C					HCW-P				
	X	DP	CV	Min	Max	X	DP	CV	Min	Max
Vertical	2.73	0.01	0.06	2.72	2.73	2.73	0.01	0.05	2.72	2.73
Horizontal	2.73	0.01	0.06	2.72	2.73	2.73	0.01	0.06	2.72	2.73

a *X*: average (mm d^–1^); SD: standard deviation; Max: maximum (mm d^–1^); Min: minimum (mm d^–1^); CV: coefficient of variation (%); HCW-C: hybrid constructed wetland control system; HCW-P: hybrid constructed wetland subsurface drainage system

Evapotranspiration in HCW-C (2.12 mm d^–1^) was significantly lower (p-value ≤ 0.05) than in HCW-P (2.58 mm d^–1^) during step I. Planted systems usually present greater water loss, as demonstrated by Vega De Lille,^59^ because of the combination of superficial water evaporation and plant transpiration. In addition, step I was conducted during the summer, which is favorable for macrophyte reproduction.^60^ Thus, the increase in plant biomass may also influence the increase in the level of evapotranspiration. However, during step II (autumn), no significant difference (p-value = 0.3063) was verified between HCWs (2.73 mm d^–1^). Mild temperatures (10–22 °C) and high humidity (80.5%) characterized the weather during this period.

Vega De Lille et al.^59^ also reported the effects of season on evapotranspiration, showing increased evapotranspiration rates in the hottest and driest months of the year, considering the tropical conditions. Papaevangelou et al.^40^ achieved evapotranspiration rates between 5.46 mm d^–1^ during winter (average temperature 15 °C) and 5.76 mm d^–1^ during summer (average temperature 27 °C). In contrast, Gangnon et al.^61^ verified evapotranspiration rates of 10.3 mm d^–1^ (*Phragmites australis*), 5.9 mm d^–1^ (*Typha angustifolia*), and 3.3 mm d^–1^ (*Scirpus fluviatilis*) under temperatures ranging from 10.2 to 20.9 °C between summer and winter, and 2.8 mm d^–1^ in the nonplanted system.

Water loss can directly affect chemical and biological reactions in the systems, mainly concentrating nutrients^59^ and reducing HRT.^62^ Thus, monitoring evapotranspiration in HCWs helps understand the pollutant removal pathways in the HCW systems, another important aspect of our study.

### 3.5 Mass Balance

TP and TN balances were conducted in the HCWs for effluent, support media storage, plant uptake, and other losses according to the adapted methodology of Wu et al.^63^ during the operation (Table 6).

**Table 6:** Total Phosphorus and Total Nitrogen Mass Balances in the HCW After 264 Days of Operation^a^

Parameter	System	Influent (mg m^–2^d^–1^)	Effluent (mg m^–2^d^–1^)	Removal Effluent (%)	Plant (mg m^–2^d^–1^)	Removal Plant (%)	Other Pathways (mg m^–2^d^–1^)	Removal Other Pathways (%)
**TP**	**HCW-P**	134.89	40.39	29.94	22.57	16.73	71.93	53.33
	**HCW-C**		87.55	64.91	-	-	47.34	35.09
**TN**	**HCW-P**	848.09	476.35	56.17	16.87	1.99	354.88	41.84
	**HCW-C**		652.68	76.96	-	-	195.41	23.04

a TP: total phosphorous; NT: total nitrogen; HCW-P: hybrid constructed wetland planted. HCW-C: Hybrid constructed wetland non-planted.

TP quantification of 134.89 mg m^–2^ d^–1^ in the influent and 40.39 and 87.55 mg m^–2^ d^–1^ in the effluent indicated higher overall removal in HCW-C (64.91%) than in HCW-P (29.94%). The plant uptake of 22.57 mg m^–2^ d^–1^ represented the least removal mechanism for TP, with 16.73% efficiency in HCW-P for the overall removal. Other removal pathways, including microorganism biodegradation and adsorption onto substrate, represented 71.93 mg m^–2^ d^–1^ in HCW-P and 47.34 mg m^–2^ d^–1^ in HCW-C, which correspond to removal efficiencies of 53.33% and 35.09%, respectively.

Similar plant uptakes were noted by Baldovi et al.^64^ with a range from 14.5% to 29.1% for *E. crassipes* when treating sanitary sewage, and by Dell’Osbel et al.^54^ with 11.67% in a VFCW and 17.92% in an HFCW (VF-HF HCW), both planted with different species, treating urban wastewaters.

Regarding TN, an overall removal of 56.17% in HCW-P and 76.96% in HCW-C was determined during the operation for 848.09 mg m^–2^ d^–1^ in the influent. Plant uptake was 16.87 mg m^–2^ d^–1^, representing 1.99% of the overall removal in HCW-P. A similar result was achieved by Kumwimba et al.^57^ with TN removal of 1.57–4.52% in hybrid systems filled with bamboo-based biochar, zeolite, alum sludge, woodchip, and flexible solid packing treating wastewater. Higher values were verified by Dell’Osbel et al.^54^ with 7.36% in a VFCW and 8.08% in an HFCW, composing a VF-HF HCW treating urban wastewater.

Ammonia volatilization can be neglected in our study since the pH of the effluent varied from 7.3 to 8.0 in step I and 6.3 to 8.1 in step II for both systems, i.e., values lower than 8.0, which were suggested by Kumwimba et al.^57^ and Vymazal^19^ for disregarding this mechanism in N removal.

In our study, microbial activity and accumulation onto substrate (adsorption) were considered as other pathways following the adapted methodology of Wu et al.,^63^ probably contributed 41.84% in HCW-P and 23.04% in HCW-C, corresponding to 354.88 and 195.41 mg m^–2^ d^–1^, respectively. Kumwimba et al.^57^ obtained similar values, with 31.46–79.76% for microbial removal and 5.74–14.96% for sediment adsorption in hybrid systems treating wastewater.

Therefore, it is highly likely that biofilm assimilation via microorganisms and adsorption onto the filtering media were the main contributors to the transformation and removal of nutrients in this study, with a lesser contribution from plant uptake.

## 4 Conclusions

The hybrid constructed wetlands (HCW) system, planted with *Eichhornia crassipes* (water hyacinth), has shown promising results in treating urban streamwater. The increased hydraulic retention time (HRT) has enhanced aeration, emphasizing the potential synergy between plant presence and HRT in optimizing wastewater treatment. The efficiency evaluation of the HCW system in removing COD, nitrogen, and phosphorus has demonstrated substantial nutrient removal capabilities. Notably, the HCW-P system has exhibited higher COD removal efficiency than HCW-C, thus highlighting the significant impact of *E. crassipes* on treatment performance. The removal efficiencies of nitrogen and phosphorus were influenced by HRT and the presence of macrophytes, showcasing the importance of these factors in nutrient removal. *E. crassipes* contributed to ammonia removal, and the hybrid system has exhibited potential for nitrogen assimilation by plants and denitrification in anoxic zones.

The growth assessment of *E. crassipes* revealed a significant increase in plant density, thus emphasizing the adaptability and resilience of these macrophytes in the HCW environment. Nutrient quantification of plant biomass confirmed the role of *E. crassipes*in nutrient uptake, supporting the efficiency of the HCW system in nutrient removal.

The lower evapotranspiration rates verified could be attributed to solar radiation, humidity, and the annual season. It is worth noting that the differences in water loss between steps I and II were primarily due to the characteristics of each season, which highlights the need for comprehensive seasonal data in future studies. The mass balance suggested biofilm assimilation and adsorption as the main mechanisms for TP and TN removal rather than plant uptake.

In conclusion, the HCW system planted with *E. crassipes* effectively treated the contaminated urban streamwater, demonstrating the important capability of removing nutrients. Our results contribute to a better understanding of the synergistic effects in aquatic environments and offer practical applications for improving water and wastewater treatment processes.

## Supporting Information Available

Presentation of the HCW systems planted with *Eichhornia crassipes* and control (nonplanted), comprising a vertical unit (VF) followed by a horizontal unit (HF), both filled with sand and gravel (PDF)
