# Supplementary material for: Intensifying Nutrient Removal in Hybrid-Constructed Wetlands Treating Urban Streamwater
Source: ACS Omega. 2025 Apr 2;10(14):13943–53. doi: 10.1021/acsomega.4c10124 (PMC12004156; doi:10.1021/acsomega.4c10124)
Supplement: Supplementary file 1 — ao4c10124_si_001.pdf [file ao4c10124_si_001.pdf]

***Intensifying nutrient removal in Hybrid-Constructed Wetlands treating urban stream water***

André Gustavo Patel[a]; Débora Gonçalves Bortolini[b]; Adelania de Oliveira Souza[a]; Mateus Xavier de Lima[a]; Ana Paula Trevisan[c]; Vsevólod Mymrin[a], André Nagalli[d]; Fernando Hermes Passig[e]; Karina Querne de Carvalho[d*]

[a] Federal University of Technology – Paraná (UTFPR) - Civil Engineering Graduate Program. Deputado Heitor de Alencar Furtado St., 5000, Ecoville, Curitiba, Paraná, Brazil. Postal Code: 81.280-340. Phone number: +55 (41) 3279-4500. E-mail: andregustavopatel@gmail.com, souza.2021@alunos.utfpr.edu.br, mateusxavier.lima@gmail.com, seva6219@gmail.com

[b] Federal University of Technology – Paraná (UTFPR) – Environmental Sciences and Technology Graduate Program. Deputado Heitor de Alencar Furtado St., 5000, Ecoville, Curitiba, Paraná, Brazil. Postal Code: 81.280-340. Phone number: +55 (41) 3279-4500. E-mail: debortolini@gmail.com

[c] Western Parana State University (UNIOESTE) - Agricultural Engineering Graduate Program. Universitária St., 2069, Jardim Universitário, Cascavel, Paraná, Brazil. Postal Code: 85.819-110. Phone number: +55 (45) 3220-7239. E-mail: anapaullatrevisan@gmail.com

[d] Federal University of Technology – Paraná (UTFPR). Civil Construction Academic Department, Deputado Heitor de Alencar Furtado St., 5000, Ecoville, Curitiba, Paraná, Brazil. Postal Code: 81.280-340. Phone number: +55 (41) 3279-4500. Email: nagalli@utfpr.edu.br, kaquerne@utfpr.edu.br

[e] Federal University of Technology – Paraná (UTFPR) – Chemistry and Biology Academic Department. Deputado Heitor de Alencar Furtado St., 5000, Ecoville, Curitiba, Paraná, Brazil. Postal Code: 81280-340. Phone number: +55 (41) 3279-4500. Email: fhpassig@utfpr.edu.br

[*]Corresponding author: kaquerne@utfpr.edu.br

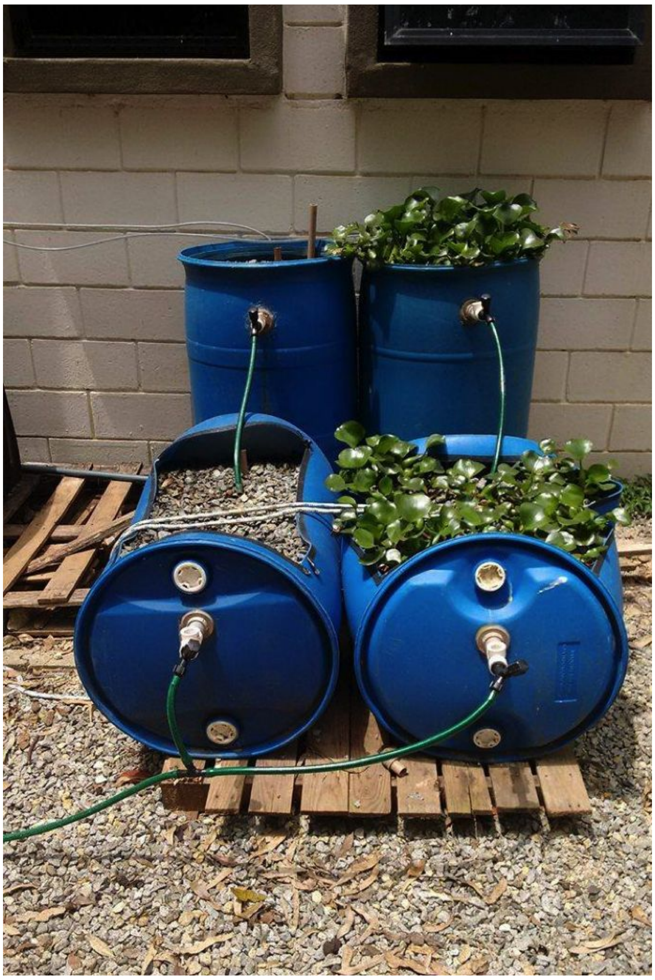

Figure S1 – Image of the HCW systems (control and planted systems)

Note: Photo taken by MSc. André Gustavo Patel
